# Nurse leaders' experiences of professional responsibility towards developing nursing competence in general wards: A qualitative study

**DOI:** 10.1111/jonm.13745

**Published:** 2022-08-10

**Authors:** Signe Gunn Julnes, Tove Myrvang, Laila Solli Reitan, Gry Rønning, Solfrid Vatne

**Affiliations:** ^1^ Department for Health and Social Care Molde University College Molde Norway; ^2^ Department of Intensive Care Medicine Molde Hospital Trust Molde Norway

**Keywords:** nurse leaders, nurses, nursing competence, qualitative research, supervisory

## Abstract

**Aim:**

To explore nurse leaders' experiences of professional responsibility to facilitate nursing competence in general wards.

**Background:**

Nurse leaders are responsible for maintaining high levels of competence among nurses to improve patient safety.

**Methods:**

Qualitative analysis was conducted between February and April 2019 using semi‐structured interview data from 12 nurse leaders in surgical and medical wards at three Norwegian hospitals.

**Results:**

Four main themes were identified: struggle to achieve nursing staff competence; focus on operational and budgetary requirements rather than professional development; demands to organize sick leaves and holiday periods; and challenges in facilitating professional development.

**Conclusion:**

Nurse leaders felt that their responsibilities were overwhelming and challenging. They witnessed more support for current administrative tasks than for the implementation of professional development. Additionally, unclear work instructions from the employer provided few opportunities to facilitate professional development. Hospital management failed to ensure quality of care and patient safety in general wards by not supporting the strengthening of nurses' professional competence and preventing turnover.

**Implications for Nursing Management:**

Management may integrate formal work instructions that clarify nurse leaders' responsibilities as professional developers, allowing nurse leaders to meet their obligation of maintaining adequate professional competence among nursing staff in general wards.

## 1 BACKGROUND

Currently, hospitals face a large number of challenges, including new patterns of diseases, rapidly evolving medical technologies, growing aging populations, and continuing budget constraints. Consequently, health professionals are required to continually develop new skills to adapt to evolutions in healthcare (North, 2020). According to the World Health Organisation (2020), nurses are the largest part of the health workforce worldwide. Nurse leaders manage large budgets, coordinate with the nursing staff in hospitals and play vital roles in administrative and executive positions. They are therefore expected to have leadership and managerial competencies, as well as substantial professional knowledge (González García et al., 2020; Kantanen et al., 2017). Nurse leaders have diverse responsibilities across finance, human resources and operations and are also responsible for developing services in accordance with official regulations and social needs (Athlin et al., 2014; Berg & Byrkjeflot, 2014).

In Norway, nurse leaders' operational responsibilities involve employing staff with appropriate competencies around the clock to ensure patient safety (Norwegian Directorate of Health, 2017; Special Health Services Act, 1999; The Norwegian Directorate of Health, 2019). Therefore, they play a vital role in motivating nursing staff to develop the required professional competence (Athlin et al., 2014; Vesterinen et al., 2013; Wikstrom & Dellve, 2009). According to the Norwegian Directorate of Health (2017), leaders must be aware of their responsibility to achieve targets, as well as offer support and flexibility needed to take care of this responsibility. The Norwegian government has stated (Meld. St. 13, 2011–2012) that leaders' competence is the totality of knowledge, skills and abilities, which enable the fulfilment of specific functions and tasks in accordance with defined expectations and goals. However, previous research shows that nurse leaders experience challenges in fulfilling their tasks. Athlin et al. (2014) highlighted the lack of congruence between nurse leaders' responsibility and authority, which obstructs achieving high quality of care and working environments. Wikstrom and Dellve (2009) described that, in practice, nurse leaders' focus is on balancing budgets and staff is seen as a cost rather than a resource.

This undermines the focus on developing necessary professional competencies. According to Armstrong et al. (2015), an enabling practice environment with supportive executive management is required to enable nursing managers to promote professional development. Kanerva et al. (2017) suggest that leadership development is a crucial element in advancing patient safety in hospitals. Reviews by Kakemam et al. (2020) and Lunden et al. (2017) emphasized that nurse leaders need evidence‐based interventions to support shared learning and create infrastructure that facilitates competence development.

In Norway, unlike many other countries, healthcare services are public (Mossialos et al., 2016). In 2002, the New Public Management reform was implemented in Norwegian public hospitals, incorporating private sector models. Four regional health enterprises were established as independent entities with their own boards and managing directors (Meld. St. 7, 2019–2020). Unified management was introduced, which led to an amendment to the Act §3–9 (Ministry of Health and Care Services, 1999). Each organizational unit was given overall responsibility for its activities, both administrative and professional, with managers at all levels; and nurses and physicians could apply for the same management positions. The goal was to establish lines of responsibility so that hospital employees knew their immediate managers. However, the Norwegian Nurses' Association believed that unified management could lead to medical domain thinking and specialization, and cause a split in the nursing service, hampering professional development and research. Studies of Norwegian hospitals have found tensions between top management levels and lower implementation levels. The current studies describe nurse leaders' experiences with various administrative tasks in general wards, but little is known about nurse leaders' experiences with their professional responsibility to facilitate competence development of the nursing staff. The aim of this study was to explore such experiences. The research question was: What were nurse leaders' experiences of their professional responsibility in terms of facilitating nursing staff competence in general wards?

## 2 METHODS

### 2.1 Settings, sample and data collection

This study was conducted in three city hospitals in Norway, with approximately 300 beds in the surgical and medical wards. The participants were registered nurses who worked as formal nurse leaders (leaders employed by the hospital to manage a general ward). The nurse leaders were chosen by a purposive sampling procedure. They were recruited from each hospital upon invitations from the hospital management after they had received written and oral information about the study from the researchers. In total, 12 of the 13 female nurse leaders working in surgical and general medical wards joined the study voluntarily.

The participants were between 35 and 65 years of age and had between 1 and more than 30 years of experience. Of the 12 participants, 10 had a degree in management or were concurrently enrolled in management education courses.

We selected a qualitative exploratory design using individual interviews Polit and Beck (2014) to capture participants' challenges regarding professional responsibilities. We developed a semi‐structured interview guide with open‐ended questions to identify nurse leaders' experiences of their professional responsibility and to facilitate competence development among the nursing staff in general wards. We asked questions such as ‘Please tell me about your professional responsibilities as a nurse leader in the general ward’, ‘How do you conduct competence development among your nursing staff?’, ‘How do you think the professional development of the nurse's competence in the general ward meets the current and future patient picture?’ and ‘How do you collaborate with the hospital management to develop strategies to promote competence development among nursing staff in general ward?’ More details were elicited by asking, ‘Can you provide an example?’ The interviews involved listening to the nurse leaders' experiences, soliciting more detailed descriptions and asking additional open‐ended questions when expected material was omitted.

The interviews conducted from February to April 2019, lasted for about 1 h, were audio‐recorded with consent and were subsequently transcribed (the average length of transcription was 15 pages). The authors conducted interviews in the meeting rooms of the participants' workplaces.

### 2.2 Data analysis

We analysed the transcribed interviews based on qualitative content analysis, inspired by Graneheim and Lundman (2004), in five steps (Table 1). First, each interview was read several times to gain a sense of the whole narrative and identify preliminary themes. Second, the text was divided into meaning units, and content from direct quotes was extracted and condensed using participants' own language. Third, the condensed meaning units were labelled with codes to organize data. Fourth, the codes were compared, similarities and differences were identified, and a structure of categories and sub‐categories was created. Finally, we summarized our comprehensive findings in an overarching category. After the analysis was conducted, the researchers agreed that the data were diverse and of quality to answer the aim of the study.

**TABLE 1:** Example of the analysis process

Meaning units	Condensed meaning unit	Coding	Sub‐category	Category
It is a confusing and demanding section to achieve competence that consists of five different patient groups: vascular surgery, gynaecology, urology, ear‐nose‐throat and eye‐care (Participant 1)	It is confusing and demanding to achieve competence since we have many different patient groups	Dividing into different patient groups makes it challenging to have an overview of necessary professional knowledge	Demanding and challenging to cover enough competence due many different patient groups	Struggle to achieve nursing staff competence
You feel that the main work here is operations management, calling to cover round‐the‐clock shifts, etc. so I say these duties have nothing to do with professional development. There are no formal policies from employers about professional development, and when we ask about the same, all we hear is that it is the budget that matters, You save nothing by going understaffed… on the contrary (Participant 6)	The main work is operations management to cover the shift; these duties have nothing to do with professional development; there are no formal policies from employers, all that matters is the budget	No formal policies about professional development Focus on shift and budget	Main responsibility is to cover shift and manage the budget	Focus on operational and budgetary requirements rather than professional development
I use 30%–40% of my working time as a nurse leader to actually cover day and night shifts, but due to sick leaves and other reasons, these plans do not work (Participant 11)	Using 30%–40% of my working time to cover day and night shifts, but due to sick leaves and other reasons, these plans do not work	Spending lot of time covering shifts when understaffed	Do not succeed to acquire required competence	Demands to maintain competence during sick leaves and holiday periods
It is difficult to have enough time … if we had a professional nurse hired for competence development. A lot could have been improved, for example, they could have performed patient safety programmes (Participant 10)	A professional nurse hired for competence development, a lot could have been improved. For example, they could have performed patient safety programmes	To collaborate with a hired professional nurse could improve competence	Lack of opportunities to improve professional competence	Challenges in facilitating professional development

### 2.3 Ethical considerations

This study was approved by the Norwegian Social Science Data Services (NSD) 59402 and conducted according to the current ethical guidelines of the World Medical Association (2000). All data were handled confidentially by storing audio material and transcriptions on an external hard drive in a locked cabinet Polit and Beck (2014) and deleting data in accordance with NSD guidelines. The research board of the regional health authorities approved this project. The participants received written and oral information about their participation and were assured of anonymity, confidentiality, voluntary participation and freedom to withdraw. All participants provided written informed consent.

## 3 RESULTS

### 3.1 Struggle to achieve nursing staff competence

Several participants reported the need for a higher level of competence to deal with increasingly complex patient cases and to improve the quality of service. They found the division of the general ward into different patient groups challenging and expressed that employees needed to be competent enough to manage the full spectrum of patient diagnoses. A participant stated: ‘Management is confusing and demanding to achieve competence since we have five different patient groups, such as vascular surgery, gynecology, urology, ear‐nose‐throat, and eye‐care’ (1). Some also reported that they felt that nurses did not consider professional development, which was frustrating because they struggled to improve competence in the ward. One participant said, ‘The most important and difficult thing is to develop the capacity for change. We need to prepare for the future, so we need to start this process now’ (10). Another said, ‘We encourage nurses to take higher clinical education, but then they move on to special units’ (2). Several participants found it unsuitable to employ nurses based on a specialization, as comprehensive education is essential in the general ward. Hence, even though participants had enough employees, they needed to hire extra nurses to cover different professional fields.

### 3.2 Focus on operational and budgetary requirements rather than professional development

Participants reported a lack of management guidelines and clarity on how to take responsibility for professional duties. They also faced ambiguous demands for ensuring the quality of their nurses' competency, for which they felt greatly responsible. One participant said, ‘There are barely any clear requirements for eligible competence, so I feel that I am solely responsible for determining the same’. Another stated that, ‘The requirements for me as a leader are not clear, but I feel responsible for ensuring that those who work with me possess the required skills’ (9).

Even though the management did not provide support regarding employees' capacity assessment, they did pressurize participants to oversee daily operations and comply with financial limits. For instance, one participant said, ‘The main work here is operations management, calling to cover round‐the‐clock shifts, etc. These duties have nothing to do with professional development. There are no formal policies from employers about professional development, and when we ask about the same, all we hear is that it is the budget that matters’ (6).

### 3.3 Demands to maintain competence during sick leaves and holiday periods

Employee management issues, such as covering sick leaves and vacations, and subsequent understaffing, were common challenges faced by the participants, which they described as being demanding. Participants spent a lot of time covering shifts while ensuring that employees represented a variety of professional skills, without succeeding. One participant said, ‘I use 30‐40% of my working time to cover day and night shifts, but due to sick leaves and other reasons, these plans do not work’ (11). Similarly, another stated, ‘I sit down to call for substitutes and I go home with a guilty conscience, because I doubt if they have the required competence’ (9). Likewise, a participant claimed, ‘It is a challenge when half the nurses go on vacation, as it is very difficult to replace them’ (3), whereas another said, ‘If nurses with lower competence are hired, working becomes harder for the remaining employees’ (10). Further, a participant said, ‘We are generally able to obtain enough resources as there are many students who want a job. However, nursing coverage drops drastically during summer vacations’ (7).

### 3.4 Challenges in facilitating professional development

Several participants mentioned that facilitating nurses' professional development was an important responsibility, and some had even integrated permanent professional development days for this purpose. They reported that it was important to motivate nurses to consider competence development, as one participant said, ‘If you do not get professional input, you may lose motivation’ (3). Participants described a desire to meet nurses' expressed needs for professional development and even offered to schedule an hour of professional meetings with them each week. Although these offers were met positively, most participants stated that they had stopped offering them because nurses did not prioritize attending them. One participant disclosed, ‘Those who work afternoon shifts forget the meetings, and it has become impossible to host them’ (11).

To support new hires, participants offered them 3 days of training and requested experienced nurses to provide additional guidance. However, participants found it challenging to organize internal or external courses for hires, as they had to prioritize daily activities. One participant said, ‘I would like to have a greater budget to send nurses to courses, but I must also ensure that there are enough competent nurses available on rotation. This is prioritized over professional development’ (9). Almost all participants wanted the management to hire professional nurses responsible for competence development. However, only a few had been granted such nurses, but not in sufficient numbers. A participant said, ‘If we had a professional nurse hired for competence development, a lot could have been improved. For example, they could have performed patient safety programs’ (1).

Some participants stated that it was a challenge to motivate experienced nurses to take over new tasks that required developing professional skills. As one said, ‘Many nurses with long careers work the same routines, making it difficult for them to understand the value of continuous professional development. What I have learned is that we all see this issue differently and do not have the same commitment toward change. So, when you must push people in the direction you want them to go, you eventually give up’ (11).

However, participants also reported that a few professional development days were used to teach mandatory exercises using online portals. These included infection control, cardiopulmonary resuscitation and fire protection.

Participants revealed that mandatory online courses with national evidence‐based clinical procedures provided guidelines for performing specific nursing responsibilities. Further, almost all participant leaders described online courses as tools for acquiring professional knowledge and opportunities for professional advancement. Several participants mentioned that these courses reassured them that their employees had undergone necessary trainings. Others declared that they viewed these courses as mandatory management tools rather than professional development opportunities. As a participant said, ‘It does not contribute to professional development, but to the fact that you must stay updated on current procedures’ (5).

## 4 DISCUSSION

The aim of this study was to explore nurse leaders' experiences of their professional responsibility to facilitate nursing staff competence in general wards. Our results indicate that the nurse leaders meet several barriers when facilitating professional competence development in general wards.

### 4.1 Ensuring competence development in general wards

Participants' descriptions of their responsibility in competence development provided an overview of the need for developing nursing competence in general wards regarding increasing complicated patient cases. They felt obligated to provide both generalist and specialist competencies. One finding was that participants had to hire extra nurses to bridge competence gaps according to patients' illnesses and needs. This finding is similar to that of Sibbald and Kothari (2015), that is, nurse leaders attempted to anticipate and promote competence, but competence development often was focused on daily management of knowledge and ‘putting out fires’. According to Lunden et al. (2019), knowledge development will occur on an ad hoc basis unless it is supported by clear structure and sufficient managerial support. They concluded that knowledge management in nursing is a complex task that requires a command of different kinds of management of related leadership styles and competence. This aligns with a finding from the present study, which discovered that in some cases, nurse leaders struggle with the requirements of professional competencies, because it was impossible to be skilled in all fields and they did not know how to solve these challenges in the long term. Another finding from our study indicated that nurses disappeared from the general ward when they completed the specialist education. This may be attributed to better job prospects. According to Moland and Bråthen (2021), nursing leaders must think innovatively to maintain competence in the general ward, such as offering specialist nurses to work in combined positions in outpatient clinics and general wards with the same field area so that competence can be maintained. The benefit is that patients meet the same nurses at admission and at outpatient visits, and the competence is strengthened on weekends. These combined positions can provide good synergies professionally and provide good coherence in the patient process. The use of combined positions can contribute to less vulnerable operations in the general ward because there are more employees available with equal competence. Combined positions will enable the solution of structural challenges by offering challenging tasks and retaining employees in the general ward and outpatient clinic. To succeed with combined positions, employees in the workplace must know tasks, routines and colleagues and, thus, do a good job and gain mastery (Moland & Bråthen, 2021).

### 4.2 Facilitating nurses' all‐round competence development

Our findings revealed that new employees receive 3 days of training and regular mentoring from nurses, but not as formal requirements, and the only criterion for mentoring was having nursing experience. According to a study by (Wei et al., 2019), one‐to‐one mentoring is an important factor in supporting the personal and professional development of new graduate nurses. Our participants had great faith in mentor training and saw it as quality assurance, claiming that support from experienced nurses is perceived positively, prevents stress and improves working environments (Chen et al., 2011). However, Chen et al. (2011) also found that providing mentoring can be laborious and burned‐out mentors can negatively impact newly hired nurses. Identifying, addressing and monitoring clinical burnout in nurses are important duties of nursing leadership (Kelly et al., 2019). In our study, nurse leaders reported struggling with nurses who were unwilling to take up new tasks or develop new skills. They also became frustrated when nurses undertook further education and left for better prospects.

Based on these findings, our impression is that nurse leaders have few opportunities and insufficient investment to provide professional development, which is not prioritized by the management. Nurse leaders only had 4 days a year to offer professional training, which often included repetitions of procedural aspects; there were few opportunities for offering other courses; and weekly development meetings were often cancelled. Even though nurse leaders desired to facilitate competence development, only a few of them had employed professional nurses to collaborate on such projects. According to Chen et al. (2011), competence development is important to job retention, especially among nurses. Lunden et al. (2017) emphasized that supported and collaborative learning and information sharing are required to develop competence.

### 4.3 Conflict between responsibility of professional development and budgetary constraints

Overall, participants reported experiencing pressure to perform their financial, professional, operational and human resource responsibilities. Particularly, their biggest challenge was that sick leaves and vacations often led to understaffing of competent staff and hiring of inadequate personnel which led to guilty consciences. This finding is supported by Armstrong et al. (2015) who highlighted that lacking healthcare employees and suboptimal performances disrupted nurse leaders' responsibilities and hindered other management tasks. This finding also aligns with other studies (Orvik et al., 2015; Orvik & Axelsson, 2012), who found that nurse leaders were conflicted between demands for both quality and efficiency.

Haahr et al. (2020) found that nurses have a holistic approach to their work. This can be linked to the ICN Code of Ethics for Nurses, which provides guidelines for accountability to society, patients, other staff and themselves. Our findings indicate that systemic requirements for cost‐effectiveness do not increase efficiency, and negatively impact professional development, hindering patient safety. Hence, nurse leaders are conflicted between the desire to improve professional competence and quality while being cost efficient.

According to López‐Medina et al. (2022), it is important to provide task‐oriented information such as role and goal clarity. Managers have the power to either support or hinder employees' professional competence and are hence linked to organizational performance. The most important obstacle to professional development in this study was the aforementioned conflict between budgetary requirements and competence development. This has been found in other international studies (Athlin et al., 2014; Orvik & Axelsson, 2012).

Another important finding was that time constraints negatively impacted leaders' contribution to competence development. Administrative routines, in particular, became obstacles towards fostering professional skills. Wikstrom and Dellve (2009) also found that administrative tasks are perceived as time consuming and stressful. After unified management was introduced in public hospitals in Norway, the Norwegian Nurses' Association (2018) claimed that nursing services did not have clear management lines, increasing administrative tasks. Further, the enterprise model fragmented nursing services and restrained professional development and collaboration, threatening patient safety (Norwegian Nurses' Association, 2018).

Norwegian hospitals are still under ministerial control. Although they are organized as state enterprises, physicians are tasked with gatekeeping services, and challenging this power is difficult (Berg & Byrkjeflot, 2014). This may be one of the reasons why nurse leaders do not have the authority to prioritize important areas of professional development. This explanation has been proposed by Vasset et al. (2021), on finding that nurses in Norway have less formal leadership power after structural changes in organizations. However, because healthcare professionals' moral duties require them to be critical of their employers' directives if they are harmful, nurse leaders' inputs toward improving patient care quality and safety in general ward management must be considered even against hierarchical authorities (Stenehjem, 2016). According to Walsh et al. (2019), leaders play a key role in the success of any organization, and there is value in understanding the competencies that affect leadership effectiveness. Although most nurse leaders in our study had received higher management education, they could not come up with innovative solutions to reduce turnover and maintain competence in general wards. Hafsteinsdóttir et al. (2019) argued that nurse leaders should be more proactive and ensure involvement and interprofessional collaboration. Strategies to reduce turnover and increase professional development may be improved by shared governance programmes with physicians that give nurses an empowered voice in scheduling, workflows and hospital policies. Nursing leaders should also establish relationships across general wards and hospitals within their respective regional health authorities in order to work towards a targeted solution to improve professional development. According to West et al. (2014), organizations cannot work in isolation to achieve the best possible care; their cultures need to be conducive to interdependent working within and across the system. Our findings suggest that nurse leaders have few opportunities in the organizational structure to increase and maintain their nursing staff's competence to promote patient safety in general wards. The hospital's management should accept responsibility for being efficient with the nurse leaders' competence and skills, as well as making the necessary changes to combat health system challenges.

### 4.4 Strengths and limitations

The trustworthiness of our findings is strengthened by our consistent adherence with the guidelines of qualitative analysis, as well as its corroboration with other research findings. However, because our data were collected from only 12 participants from regional hospitals, our findings cannot be generalized across regions and organizations.

## 5 CONCLUSIONS

Nurse leaders perceived their responsibilities as overwhelming and challenging. Initiatives from the hospital management regarding appropriate competence development guidelines would be essential in facilitating nurse leaders' capacity beyond monitoring budgets and staff. Nursing leaders must stay informed about innovations that may have an impact on the organization's goal of reducing turnovers and improving workflow in institutionalized nursing care. This study identified critical issues that should be addressed in future research.

## 6 IMPLICATIONS FOR NURSING MANAGEMENT

Hospitals should formulate clear work guidelines to strengthen nurse leaders' abilities to assess and maintain nurses' professional competence in general wards. Nurse leaders should be allocated more resources to conduct their duties, such as nurses to manage professional development and assistants to perform administrative tasks such as managing shifts, sick leaves and holidays.

## CONFLICT OF INTEREST

The authors declare that they have no competing interests.

## ETHICS STATEMENT

This study was approved by the Norwegian Social Science Data Services (NSD) (number 59402) and conducted according to the current ethical guidelines of the World Medical Association (2000). All data were handled confidentially by storing audio material and transcriptions on an external hard drive in a locked cabinet Polit and Beck (2014) and deleting data in accordance with NSD guidelines. The research board of the regional health authorities approved this project. The participants received written and oral information about their participation and were assured of anonymity, confidentiality, voluntary participation and freedom to withdraw. All participants provided written informed consent.

## Data Availability

Research data are not shared.
